# Editorial: Nutrients and brain: from bench to the bedside

**DOI:** 10.3389/fnhum.2023.1184468

**Published:** 2023-05-05

**Authors:** Mariagiovanna Cantone, Giulio Geraci, Justyna Godos, Giuseppe Lanza

**Affiliations:** ^1^Neurology Unit, Policlinico University Hospital “G. Rodolico-San Marco”, Catania, Italy; ^2^Internal Medicine Unit, Sant'Elia Hospital, ASP Caltanissetta, Caltanissetta, Italy; ^3^Department of Biomedical and Biotechnological Sciences, University of Catania, Catania, Italy; ^4^Department of Surgery and Medical-Surgical Specialties, University of Catania, Catania, Italy; ^5^Clinical Neurophysiology Research Unit, Oasi Research Institute–IRCCS, Troina, Italy; ^6^Center for Human Nutrition and Mediterranean Foods (NUTREA), University of Catania, Catania, Italy

**Keywords:** Mediterranean diet, brain aging, cerebrovascular disease, neuroprotection, age-related cognitive disorders

An increasing attention toward the beneficial effects of diet and dietary components on brain health, including neurodevelopment, mental functioning, and brain aging, has been recently noted. Although epidemiological studies have still not reached a definitive conclusion regarding a protective effect of nutrition, several dietary components are traditionally considered as preventing factors for several age-related disorders, such as cardio-cerebrovascular diseases and cognitive complaints (Fisicaro et al., 2021a, 2022; Caruso et al., 2022a).

Emerging evidence suggests that adherence to traditional dietary patterns rich in fruits, vegetables, whole grains, legumes, nuts, and fish, such as in the Mediterranean diet, protects against cognitive decline of vascular or degenerative origin and age-related memory impairment (Panza et al., 2004; Dinu et al., 2018; Shannon et al., 2023), thus highlighting the relevance of diet among the interventions possibly preventing or slowing down dementia. Recently, an anatomopathological study has clearly shown that the Mediterranean diet was associated with less *post mortem* Alzheimer's disease (AD) pathology, primarily the beta-amyloid load. Namely, among dietary components, green leafy vegetables inversely correlated with AD pathology (Agarwal et al., 2023). Indeed, the neuroprotective effects of Mediterranean diet are mainly attributed to its richness in bioactive compounds, such as omega-3 fatty acids and polyphenols (Godos et al., 2020; Caruso et al., 2022b; Kurowska et al., 2023). However, although data suggests their role on pathophysiology and progression of brain disorders, their relationship with clinical symptoms and neuropsychological or behavioral profile in the context of neurodegenerative or cerebrovascular diseases remains to be elucidated.

In this scenario, the need for an early diagnosis of neurodegenerative disorders has focused on the identification of laboratory and instrumental markers of disease process and progression, although there is still no consensus regarding a dose-response effect on clinical features, cognition, mood, or other neurological manifestations. Nevertheless, the identification of new and possibly modifiable protective or risk factors will be of crucial importance for patients, clinicians, and researchers. In this context, novel research data will represent the scientific platform for the development of new dietary approaches, such as tailored age-correlated diets or anti-aging diets. Moreover, findings from complex anti-aging interventions will help the transition from preclinical to clinical settings, thus also providing hints for nutritional guidelines for older adults, both in physiological (normal brain aging) and pathological conditions (cognitive decline).

With this in mind, this Research Topic collected both original research and review articles addressing recent evidence on the effects of diet or dietary components on brain health and neurological or neuropsychiatric disorders, including findings from neuroimaging, electrophysiology, and neurosonology.

Hidese et al. investigated the relationship between the body-mass index (BMI) and both gray matter volume and white matter parameters. In particular, the authors reported different measures derived from a 3 T magnetic resonance imaging (MRI) of 382 mentally healthy right-handed subjects of both sexes, who covered a wide range of BMI values. From the voxel-based morphometry, they found an inverse U relationship between brain structural parameters and BMI, as well as a significant positive correlation between BMI and total white matter volume. The diffusion tensor imaging data showed a positive correlation between the right superior longitudinal fasciculus mean diffusivity and BMI. Overall, the authors confirmed previous results about reduced gray matter region volumes in overweight or obese individuals. More interestingly, however, they demonstrated for the first time that gray matter volume was reduced also in underweight individuals, thus highlighting as the body weight strongly influenced the gray matter volume. As such, an adequate control and monitoring of the body weight may be of beneficial for a healthy brain. More recently, new indices of adiposity, that seem to go beyond the limits of BMI, have been shown to correlate better with cardiovascular risk (Mulè et al., 2017; Geraci et al., 2018, 2019) and in the future they could be investigated in relation to cerebrovascular manifestations or other brain disorders.

Fisicaro et al. explored the cerebral hemodynamics to transcranial Doppler sonography (TCD) in 15 newly diagnosed celiac disease (CD) patients at an early stage of the disease and without clear neurological symptoms. Overall, the authors showed subtle changes of cerebral vasomotor reactivity in the posterior circulation in CD patients, which might be considered to be in line with the subclinical brain cortex involvement observed to transcranial magnetic stimulation in the same cohort of patients (Pennisi et al., 2017; Lanza et al., 2018; Fisicaro et al., 2021b).

In the manuscript by Infortuna et al., the authors explored the functional connectivity to TMS between the olfactory and motor cortex by assessing changes in corticospinal excitability following actual and imagined exposure to the smell of bergamot oil or saline. The authors considered the Bergamot suitable for both cosmetic and food industry, as well as for its therapeutic potential for psychiatric disorders. The results indicated that corticospinal excitability changed after both perception and imagery of a pleasant odor, whereas the correlation analysis showed an association with neuroticism personality trait and general olfactory imagery ability. The authors concluded that both perception of a pleasant odor and its olfactory imagery modulated motor cortex excitability and that an enhanced brain activation was affected by specific individual characteristics.

Finally, in the last years, several researchers evaluated interventions for impaired bioenergetics in chronic inflammatory demyelinating disease of the central nervous system, such as Multiple Sclerosis (MS). To this end, Ostojic explored the potential as a nutritional adjuvant to MS therapy of the guanidinoacetic acid (GAA), an experimental nutrient that is a direct natural precursor of creatine, thus playing a key role in high-energy phosphate metabolism. Its exogenous administration increases tissue levels of creatine across the human brain and data from preclinical trials seem to suggest a neuroprotective action by enhancing the oligodendrocyte survival after demyelinating injury. In his mini-review, the author summarized the studies evaluating GAA effectiveness in MS, explored mechanisms of GAA action, and discussed the challenges of using dietary GAA as an element of MS therapy. Interestingly, no clinical trial has evaluated the effects of GAA alone in MS, whereas it appeared to be effective when co-administered with other nutrients and/or therapeutic exercise.

Concluding, many practical questions regarding the design of diets to specifically improve brain function, such as type, frequency, and amount of nutrients, remain to be answered. Supported by this Research Topic, we are beginning to uncover the basic principles that are involved in the actions of the so-called “brain foods for mental fitness”. Incorporating this knowledge into the design of novel treatments will be vital to fight neurological and mental disorders.

## Author contributions

MC, GG, and GL draft the manuscript and conduct the analysis of data. GL and JG revise the manuscript critically for important intellectual content. All authors approved the version of the manuscript to be published.
